# Unexpected case of Graves` disease induced myocarditis: a case report

**DOI:** 10.1097/XCE.0000000000000297

**Published:** 2024-01-10

**Authors:** Widya Safitri, Dian Yaniarti Hasanah, Celly Anantaria Atmadikoesoemah, Andi Mahavira

**Affiliations:** a Division of Clinical Cardiology, Department of Cardiology and Vascular Medicine, Faculty of Medicine, University of Indonesia, National Cardiovascular Center Harapan Kita; b Division of Nuclear Cardiology and Cardiovascular Imaging, Department of Cardiology and Vascular Medicine, Faculty of Medicine, University of Indonesia, National Cardiovascular Center Harapan Kita, Jakarta; c Depok Regional Hospital, West Java, Indonesia

**Keywords:** acute coronary syndrome, Graves` disease, heart failure, myocarditis

## Abstract

Myocarditis due to Graves` disease is rare and has a clinical presentation that mimics acute coronary syndrome. In this case report, a 50-year-old woman was admitted with a clinical presentation of very high-risk non-ST segment elevation myocardial infarction, new-onset atrial fibrillation, and acute heart failure. Normal coronary angiography and the presence of intra-myocardial late gadolinium enhancement based on cardiac MRI led to the diagnosis of myocarditis. The presence of thyroid nodules and elevated thyrotropin receptor antibodies indicated Graves` disease as the underlying cause of myocarditis. Management using Propylthiouracil and the guideline-directed medical therapy for heart failure successfully improved the patient’s condition. Early diagnosis, effective care, and adequate knowledge of the relationship between hyperthyroidism and myocarditis, improve outcomes in Graves’ disease-induced myocarditis.

## Introduction

Myocarditis is an inflammatory disease of the myocardium with a wide range of clinical presentations. Myocarditis often mimics acute coronary syndrome (ACS), making the diagnosis challenging. Based on a systematic analysis of the global burden of disease by Wang X *et al*., the number of new cases of myocarditis in 2017 was 3 071 000. It was a 59.6% increase from 1990. Between 1990 and 2017 mortality and disability-adjusted life years (DALYs) for myocarditis globally have also increased [1]. There are various etiologies of myocarditis such as viruses, bacteria, parasites, toxins, drugs, or internal triggers (i.e. autoimmune activation against self-antigens).

Graves’ disease is an autoimmune disease with hyperthyroidism as the common feature. Graves’ disease may have some cardiac manifestations, such as arrhythmia, cardiomyopathy, and heart failure [2]. Myocarditis is a rare complication of autoimmune hyperthyroidism. According to Ammirati *et al*., hyperthyroidism-related autoimmune myocarditis was rare, with an incidence of approximately 0.5% [3]. Herein, we present a case of a 50-year-old female with Graves’ disease-induced myocarditis.

### Case illustration

A 50-year-old female was admitted to our emergency unit with a chief complaint of worsening chest pain a day before admission. She felt prolonged left chest discomfort, with a heavy sensation. Radiating pain, excessive sweating, or nausea were absent. Cardiovascular risk factors, such as hypertension, dyslipidemia, and menopause were reported. Previous medical history showed the presence of a lump at the front of the neck identified 2 years prior. No tremors, palpitations, heat intolerance, or weight loss were reported. A week before admission the patient was diagnosed with urinary tract infection and was treated with Ciprofloxacin for 2 days.

Physical examination found her to be compos mentis, with a blood pressure of 146/80 mmHg, heart rate of 122 beats per minute, oxygen saturation of 98% on room air, and body temperature of 38 °C. No exophthalmos. The conjunctiva was pale. A mobile, 3 cm in diameter, nodule, was detected on the thyroid palpation. The first and second heart sounds were normal without any murmur, rubs or gallop. Rales were present in the basal surface of the lung.

**Fig. 1:**
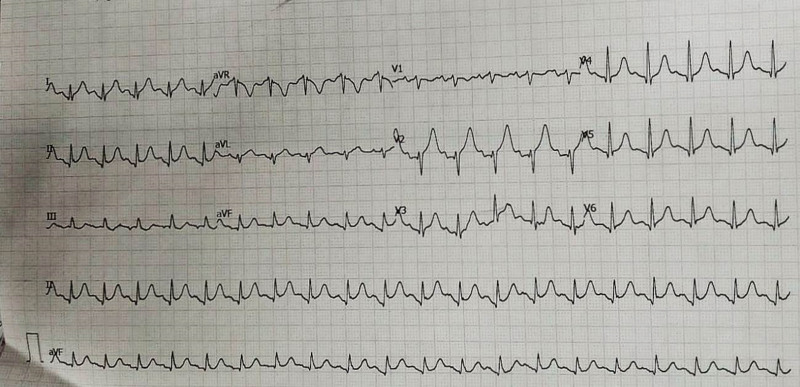
ECG at first admission in the emergency department.

Electrocardiogram (ECG) showed sinus tachycardia, normal axis, normal P waves, normal PR interval, and Spodick’s sign (Fig. 1). Laboratory assessment showed an elevated Troponin I level of 1.26 ng/ml and an elevated CK-MB level of 7.8 ng/ml. Hemoglobin level was 10.9 g/dl, normal red blood cells index, normal leucocyte count, elevated erythrocyte sedimentation rate, and elevated C-Reactive Protein level. Renal function, blood glucose and urinalysis showed normal results. Echocardiogram showed a reduction in left ventricular ejection fraction of 44%, with global hypokinetic. The previous echocardiogram a year before showed a normal ejection fraction of 67% and global normal wall motion.

Graves’ disease was considered to be the underlying cause based on the finding of a thyroid nodule. Several additional examinations, such as thyroid ultrasound and thyroid function test, were carried out. Thyroid function test showed a high free T4 level of 2.41 ng/dl (normal range: 0.93–1.7 ng/dl), very low TSH level < 0.005 µIU/ml (normal range: 0.27–4.2 µIU/ml) and high thyrotropin receptor antibody (TRAb) level of 14.21 IU/L (normal range: < 1.75 IU/L).

Based on the clinical presentation and the test results, the patient was diagnosed with very high-risk non-ST elevation myocardial infarction (NSTEMI), acute heart failure in ACS and hyperthyroidism due to Graves’ disease. Double anti-platelets (Clopidogrel and Aspirin), Enoxaparin injection, Candesartan, Statin and intravenous Furosemide were administered. On the second day of hospitalization, chest pain and shortness of breath remained, without any ST-T segment changes or ECG evolution indicating ischemia. Based on the diagnosis, an early invasive strategy was carried out on the second day of hospitalization. Unexpectedly, the coronary angiogram revealed normal coronary arteries. Given this finding, myocarditis was suspected. Loop diuretic, Candesartan, and Bisoprolol were additionally administered afterwards.

**Fig. 2:**
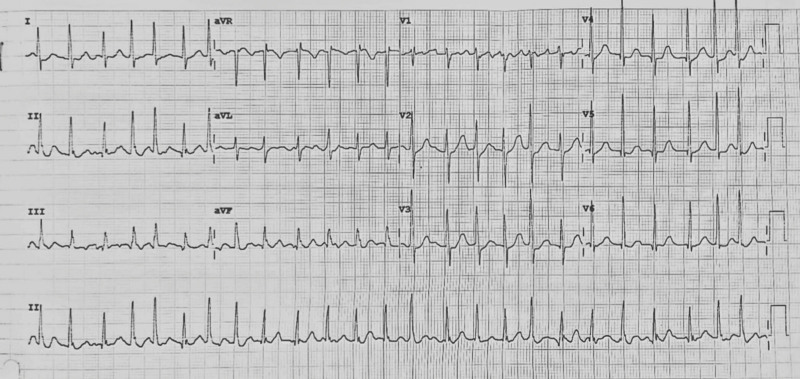
New-onset atrial fibrillation on third day of hospitalization.

On the third day, the patient had paroxysmal atrial fibrillation (Fig. 2). Propylthiouracil (PTU) (100 mg, three times a day) was furthermore given to treat hyperthyroidism. Atrial fibrillation was thereafter converted to sinus rhythm. On the fifth day of hospitalization, the patient had no symptoms and was discharged on the sixth day with Warfarin as an additional treatment.

**Fig. 3:**
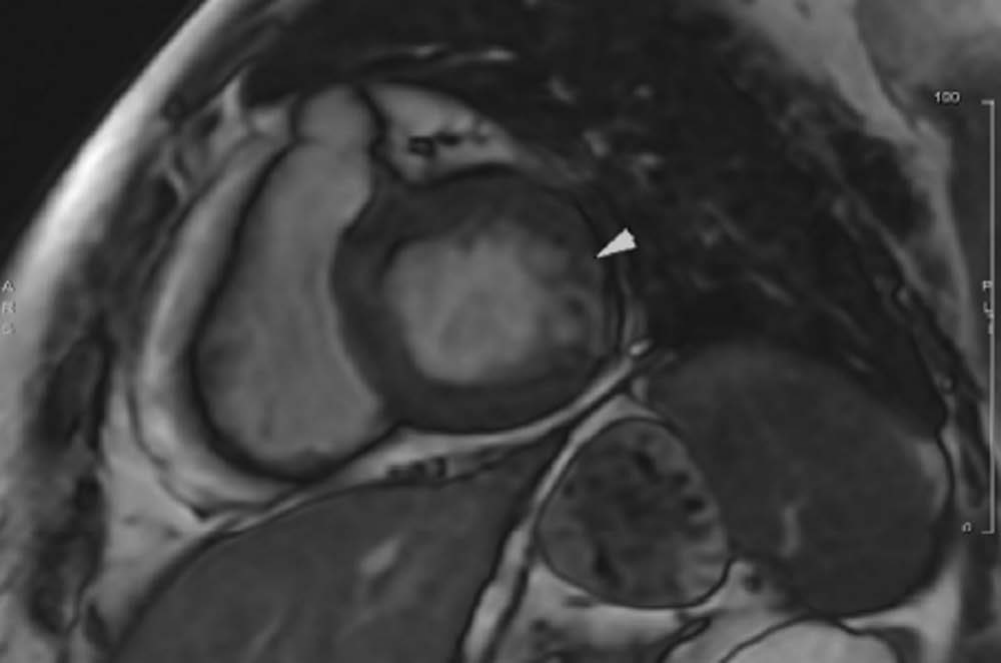
Cardiac MRI at 3 weeks after hospitalization showed patchy intramyocardial late gadolinium enhancement at basal anterolateral.

Three weeks after hospitalization, cardiac MRI was performed to evaluate the etiology of the disease. Cardiac MRI showed patchy intramyocardial late gadolinium enhancement at basal anterolateral, increased T1 relaxation time and normal T2 relaxation time. Normal left and right ventricle contractility, global normokinetic left and right ventricle, and normal valves (Fig. 3). According to the cardiac MRI findings, the patient had healed myocarditis. When this examination was carried out, the patient still continued to take Candesartan, Bisoprolol, Warfarin and Furosemide. However, PTU was discontinued on her own because she felt better.

Two months later, the patient was readmitted due to shortness of breath and atrial fibrillation with rapid ventricular response. Intravenous Digoxin and PTU, alongside a guideline-directed medical therapy for heart failure, were applied. The patient was discharged on the third day. She was able to return to normal activities without functional limitations.

## Discussion

Myocarditis is an inflammatory disease of myocardium. It can be caused by infectious or noninfectious diseases. Myocarditis may present as mild symptoms (e.g. chest pain, palpitations with transient ECG changes) or as life-threatening cardiogenic shock and ventricular arrhythmia [4]. Myocarditis should be considered in patients with clinical signs and symptoms of acute myocardial infarction, elevated cardiac biomarkers (e.g. troponin), ECG suggesting acute myocardial injury, arrhythmia, or global or regional abnormalities of LV systolic function, with normal coronary angiogram. Particularly when the clinical findings are new and unexplained, myocarditis should be suspected [5].

In our case, the patient came with severe chest pain and shortness of breath a day before admission. The presence of chest pain, cardiovascular risk factors (i.e. hypertension and menopause), and increased troponin levels support the diagnosis of NSTEMI. However, considering the chest pain and shortness of breath remained persistent, no ECG changes indicating ischemia, and normal coronary angiogram results, myocarditis is more likely to be the working diagnosis.

Endomyocardial biopsy (EMB) has traditionally been the gold standard test for myocarditis. EMB diagnosis value is based on Dallas histopathology criteria and is highly recommended for patients with life-threatening clinical presentations. Nonetheless, not all hospitals have EMB facilities and not all patients are willing to be assessed using this test [4]. Recently, cardiovascular magnetic resonance (CMR) has emerged as an important technique in the evaluation of cardiovascular disease. CMR has some advantages, including non-radiation, non-invasive, high-resolution features, and can be coupled with some new technologies. Compared to EMB, CMR was considered to be more convenient and accurate in establishing myocarditis diagnosis [6].

CMR establishes the diagnosis of myocarditis using three types of images: T2-weighted (T2-W), early T1-weighted (EGE) images taken after 1 min and delayed enhanced images (LGE) taken after 15 min of the injection of contrast agent [7]. In a setting of acute myocarditis case, there are increased regional or global T2-weight, increased regional or global T1-weight and non-ischemic patterns in LGE images. The presence of two positive criteria is sufficient to establish the diagnosis of myocarditis. On the other hand, in myocardial infarction, CMR shows sub-endocardial myocardial perfusion defect and delayed myocardial enhancement, consistent with the distribution of the coronary arteries. In addition, the delayed enhancement usually does not go away [8]. In our case, the CMR finding of patchy intra-myocardial late gadolinium enhancement at basal anterolateral (which is inconsistent with the distribution of coronary arteries) and extremely rapid T1 relaxation time support the diagnosis of myocarditis, instead of acute myocardial infarction.

There are various etiologies of myocarditis, including autoimmune disease. Graves’ disease is an autoimmune disease that primarily affects thyroid gland. Graves’ disease is common. It affects approximately 2% of women and 0.2% of men globally (i.e. female to male ratio of 10 : 1) [9]. Diagnosis of Graves’ disease is established when the clinical presentation of hyperthyroidism, diffuse goiter, and positive TRAb or TSHR autoantibody are present [10]. The patient in our case never had any symptoms of hyperthyroidism before, but a lump in front of her neck had been felt for 2 years. Increased T4 level, very low TSH level, and the presence of TRAb support the diagnosis of Graves’ disease. Their features were similar to the case of Graves’ disease-induced myocarditis reported by Lancester *et al*. in 2019 [11].

Thyroid hormone acts in heart and affects every anatomic and physiologic component of the cardiovascular system [12]. Hyperthyroidism affects cardiovascular system through hyperdynamic, hypermetabolism, genomic, and non-genomic molecular regulation [13]. Graves’ disease is one of the most common causes of hyperthyroidism. In Graves’ disease, autoimmune reaction induces the production of anti-TSH-receptor (anti-TSH-R) autoantibodies by B-cell clones infiltrating the thyroid gland. The antibodies target the extracellular domain of TSH-receptor, leading to unregulated thyroid hyperfunction. TRAb plays a major role in the pathophysiology of Graves’ disease and its cardiovascular complications.

Pathophysiology of Graves’ disease-induced myocarditis is related to myocyte damage through hormonal effects or ion channel regulation and uncontrolled autoimmune response. Both genomic and non-genomic actions contribute to the development of Graves’ disease-induced myocarditis. This leads to a complex cascade of inflammation and continuous destruction of the heart muscle in the acute phase. In the chronic stage, fibrous repair and structural remodeling are the main changes [14].

The infiltration of inflammatory cells and the production of many inflammatory factors aggravate myocyte necrosis in Graves-induced myocarditis. In healing stage, myocyte damage is localized, and the release of inflammatory cytokines is reduced. Lymphocytes interact with macrophages. Mesenchymal reparative tissue appears and is gradually substituted by replacement fibrosis. These histopathological changes can be seen on EMB. Unfortunately, our patient refused to undergo this procedure.

Myocarditis treatment includes general measures to treat the sequelae of heart disease. The general measures involve heart failure therapy, treatment for arrhythmias, and targeted therapy for specific disorders [4]. In this case, the guideline-directed medical therapy including Candesartan, Bisoprolol, and Furosemide had been given to treat heart failure. Diagnosis of autoimmune myocarditis should be considered given that the patient had Graves’ disease and that the symptoms improved quickly after hyperthyroidism treatment was administered. One of the important steps to slow the progression of hyperthyroidism-associated myocarditis is specific treatment for thyrotoxicosis. The treatment works by reducing the production and release of thyroid hormone, reducing hormone’s side effects, and increasing the excretion of thyroid hormone. By stabilizing hemodynamic status and managing thyrotoxicosis properly, heart damage and cardiac function may be improved. However, it remains unknown if antithyroid medications alone can effectively cure hyperthyroidism-induced myocarditis [15].

## Conclusion

Myocarditis has a wide range of clinical presentations, ranging from asymptomatic to mimicking ACS, acute heart failure and malignant arrhythmia. Although rare, Graves’ disease-induced myocarditis should be suspected in female patients with cardiac symptoms, history of thyroid enlargement, and elevated thyroid hormones. Early diagnosis, effective care, and adequate knowledge of the relationship between hyperthyroidism and myocarditis are necessary in the management of Graves’ disease-induced myocarditis.

## Acknowledgements

Ethics approval: This study was carried out in accordance with the Code of Ethics of the World Medical Association (Declaration of Helsinki). Approval was granted by the Ethics Committee of National Cardiovascular Center Harapan Kita, Jakarta, Indonesia (Chairperson Prof Yoga Yuniadi) on 1 August 2023.

### Conflicts of interest

There are no conflicts of interest.
